# The Robot is Present: Creative Approaches for Artistic Expression With Robots

**DOI:** 10.3389/frobt.2021.662249

**Published:** 2021-07-29

**Authors:** Carlos Gomez Cubero, Maros Pekarik, Valeria Rizzo, Elizabeth Jochum

**Affiliations:** ^1^ Research Laboratory for Art and Technology, Department of Communication and Psychology, Aalborg University, Aalborg, Denmark; ^2^ Human Robot Interaction Laboratory, Department of Architecture, Design & Media Technology, Aalborg University, Aalborg, Denmark; ^3^ Independent Artist, Aalborg, Denmark

**Keywords:** artistic research, drawing, performance, dance, robot, creativity, human robot interaction, embodied interaction

## Abstract

There is growing interest in developing creative applications for robots, specifically robots that provide entertainment, companionship, or motivation. Identifying the hallmarks of human creativity and discerning how these processes might be replicated or assisted by robots remain open questions. Transdisciplinary collaborations between artists and engineers can offer insights into how robots might foster creativity for human artists and open up new pathways for designing interactive systems. This paper presents an exploratory research project centered on drawing with robots. Using an arts-led, practice-based methodology, we developed custom hardware and software tools to support collaborative drawing with an industrial robot. A team of artists and engineers collaborated over a 6-month period to investigate the creative potential of collaborative drawing with a robot. The exploratory project focused on identifying creative and collaborative processes in the visual arts, and later on developing tools and features that would allow robots to participate meaningfully in these processes. The outcomes include a custom interface for controlling and programming robot motion (EMCAR) and custom tools for replicating experimental techniques used in visual art. We report on the artistic and technical outcomes and identify key features of process-led (as opposed to outcome-led) approaches for designing collaborative and creative systems. We also consider the value of embodied and tangible interaction for artists working collaboratively with computational systems. Transdisciplinary research can help researchers uncover new approaches for designing interfaces for interacting with machines.

“Art does not reproduce the visible; rather, it makes visible.”—Paul Klee^1^

## 1 Introduction

The study of the relationship between human creativity and machines has fascinated artists and engineers for centuries. The earliest machines mechanically reproduced activities associated with human creativity and artistic expression: playing musical instruments, drawing, dancing, and writing (Schaffer, 1999; Riskin and Bregović, 2017). From ancient automatons to recent applications of machine learning, artists and scholars continually explore new approaches for understanding and modelling expressions of human creativity (Herath et al., 2016; Laviers and Egerstedt, 2014). Machines designed for artistic expression function as both tools for art making and sites for creatively exploring the nature of interaction and human-machine interfaces. Identifying the hallmarks of creativity and discerning whether or how these processes can be replicated or assisted by computers or robots remain open and highly contested questions (Boden, 1994; McCormack and d’Inverno, 2012; Laviers and Egerstedt, 2014). Our interest is in exploring how robots function as creative tools and catalysts for artistic expression, and using the arts to help uncover new approaches for designing interfaces for interacting with machines. This article describes an arts-led, practice-based research investigation that explores collaborative drawing between human artists and an industrial robot. Rather than starting with a predefined research question, we conducted a series of workshops to explore how an industrial robot could be a catalyst for human creativity. Our transdisciplinary research team was comprised of artists, engineers and creative technologists who worked collaboratively over a 6-month period in a series of workshops. Together, we identified creative and collaborative processes in visual art making (namely drawing and painting) and explored how a robot could participate meaningfully in those processes. This inquiry led to the design of new tools that enabled the artist to work directly with the robot through tangible interaction in real-time. The intention of these tools was not to control or produce a specific preconceived outcome, but rather to make the robot more accessible as a tool for collaborative and creative expression. The project resulted in several tangible outcomes, including a custom interface for controlling and programming robot motion (EMCAR), custom hardware for replicating experimental techniques used in visual art, and an original human-robot dance performance titled *If/Then*. We present the outcomes of our artistic research, emphasizing the systems theory models of creativity proposed by Csikszentmihalyi and Getzels (2014) and Dahlstedt (2012). We contextualize our findings in relation to other arts-engineering collaborations as a way of thinking about the relation between creativity and robotics. We try to avoid reading creativity backwards from a finished product that traces back to an initial idea or question (Ingold, 2009), choosing instead to attend closely to the creative processes and generative movements that marked our collaboration. We reflect on the improvisational and spontaneous dimensions of the process that informed the development of an interactive system. Finally, we discuss the value of transdisciplinary research teams and arts-led approaches for designing and developing collaborative and creative interactive systems.

## 2 Background

### 2.1 Drawing and Creativity

Drawing is a hallmark of human creativity and one of the oldest known forms of nonverbal communication. The caves in Lascaux and Pindral feature paintings from c.13000B.C., and traditional Indigenous rock art dates back even further (19,000 years). Earlier still, ephemeral drawing practices in sand are part of oral storytelling traditions by First Nations communities, where storytellers combine oral and gestural narration during storytelling rituals (Tafler, 2019). As an art form, drawing is widely recognized as a “natural extension of the visualisation of emotions, thoughts, and ideas” of human experience through the figurative use of line and materials (Wells, 2013, p.36). As an activity, drawing involves the physical act of an artist working with and through materials and tools to arrive at some poetic visual expression. We were interested in drawing as a way of exploring human-machine creativity. Drawings are produced through physical interaction with tools (a brush, charcoal, a stick, the hand, a computer mouse) and different materials (the canvas, oils or acrylic paints, sand, pixels on a computer screen). Drawing involves tactile and sensuous knowledge—what Tim Ingold calls textility*—*where the artist and materials engage in an artful and responsive negotiation of feeling and form. For Ingold, art works are never finished but works in progress and involve emergent processes wherein the artist uncovers possibilities by learning to “follow the materials.” In *The Textility of Making* he writes, “As practitioners, the builder, the gardener, the cook, the alchemist and the painter are not so much imposing form on matter as bringing together diverse materials and combining or redirecting their flow in the anticipation of what might emerge” (Ingold, 2009, p.94). An emergent view of art making holds that the material world is not passively subservient to human designers and offers a view of creative processes as a negotiation between the artist and materials. Ingold’s characterisation of the relationship between artist, tools, and materials invites parallels with Gilbert Simondon’s view of how humans interact with machines (Simondon, 2016). Simondon likens humans working with technical machines to a musical conductor directing musicians in performance, where the human operator acts as a coordinator or organiser of a society of technical objects, determining the tempo of performance and managing the margins of indeterminacy inherent to machines. Ingold and Simondon’s ideas about art making and human-machine interaction offer new perspectives on the relationship between human artists, machines, and creativity.

Assessing creativity in drawing usually involves an analysis of the drawing itself as evidence of some kind of poetic feeling or impulse that originates inside the artist. This limited understanding that links creativity to either an individual trait, cognitive process, or attribute of an art work has been eclipsed by systems theory models that conceive of creativity as a process between cultural (symbolic) and social forces (Csikszentmihalyi and Getzels, 2014; Csikszentmihalyi, 1998). Csikszentmihalyi observed fine art students given a drawing task and developed a systems-theory approach to describe the discovery-oriented behavior as a model for understanding creative processes. Palle Dahlstedt uses his own experiences as a music composer and improvisational performer to develop a process-based model for artistic creativity centered on the use of computational tools Dahlstedt (2012). Dalhstedt defines creative practice as an “exploration of a largely unknown space of possibilities” that can be explored through an iterative process of interaction between theoretical ideas, the attending material representations achieved through implementation, and the artist’s ongoing negotiation between these two processes (Dahlstedt, 2012, p.210). The systems theory view of creativity posits that technological tools can be more than mere instruments for art making; they can embody complex behaviors and enable new lines of thought that would not otherwise be possible. At the same time, the nature of a tool sets the constraints for the exploration. If we understand drawing as something more than mere marks on a page (Walter, 1996) and instead regard it as a creative activity predicated on processes that involve human artists working with tools and materials, we can recognize drawing as a dynamic and relational process. Following Ingold, our intention is to move past the idea of an artist imposing preconceived forms on inert matter and instead consider human-machine interaction as a “looping,” generative dialogue between the image in the artist’s mind and the tools and materials at hand. Only then can we begin recognise how tools—be it a paintbrush, a computer or a robot—can negotiate the subtle and reciprocal relationships between the artist and material and become part of the dynamic assemblage that facilitates the creative endeavour.

### 2.2 Drawing Machines

Humans and tools are continually modifying each other (Stiegler, 1998; Hayles, 2012). This is true for tools developed for utilitarian practices as well as those in service of artistic expression. N. Kathrine Hayles explains the necessity of evaluating technical objects, especially digital tools, not only according to their function but as objects deeply embedded within larger social/technical processes. Following Simonodon, Hayles refers to “technical ensembles”: processes and practices through which fabrication comes about, wherein the toolmaker herself is embedded in both the practice and also in a society in which the knowledge of how to make tools is preserved, transmitted, and developed (Hayles, 2012, p.88). The evolution of drawing machines, devices that through analogue or digital means engage in drawing with varying levels of human involvement, are good examples of technical ensembles. In their introduction to and edited collection of *The Machine As Artist*, Smith and Fol Leymarie present an historical overview of drawing machines and identify key conceptual frameworks and broader philosophical questions that drawing machines pose (Smith and Fol Leymarie, 2017). The history of drawing machines includes analogue, non programmable devices such as the pantograph and pendulum-driven harmonographs, to programmable automata capable of reproducing handwriting and drawing. Beginning in the 1960s, artists like Desmond Paul Henry and Harold Cohen pioneered the fields of machine and computer art. Henry’s works with machine-generated effects are considered forerunners to computer graphics, and Cohen’s AARON, an evolving, rule-based software program has produced numerous drawings and paintings for more than 40 years, mimicking the way that human painters work with physical materials and developing an original “style” of its own (Nake, 2012). These systems and art works intersect in compelling ways with experiments in kinetic sculpture, most notably Jean Tinguely’s spectacular drawing machines. Tinguely built kinetic sculptures that produced chaotic art works according to principles of chance and unpredictability related to the mechanical designs of the machine (Salter, 2010). Tinguely’s works were ultimately valued more for their sculptural properties than the aesthetic qualities of the drawings the machine produced, but they succeeded in exploring creative possibilities of technical ensembles.

Following Cohen’s pioneering work with AARON, numerous HCI researchers and artists working in media art used practice-led research to explore creative potential of computers for art making. Within the field of computational creativity, artists recognize the potential of software and other computer-based tools to augment their creative processes, and, following systems theory, identify those tools, methodologies and practices that can support human creativity (Mamykina et al., 2002) (Quantrill, 2002). Michael Quantrill characterizes computers as “explorers,” and uses drawing as a method of investigating human-computer integration in artistic practice without de-centering the human artist: “The idea is to use the properties of computing machines to enable forms of expression that are unique to a human-machine environment where the human is the focus, but the expression is a composite of both human and machine, in this case a computing machine environment” (Quantrill, 2002, p.218). Similarly, Oliver Bown draws on the systems theory model and Alfred Gell’s notion of primary and secondary agency in his theory of computational creativity (Bown, 2012). Digital tools, like art works, can be considered secondary agents, and hint at the possibility of nonhuman agency that reveals “a gradient of agency rather than a categorical division” (Bown, 2012, p.367). While the subject of machine agency is compelling, we are more interested in investigating robots as tools for facilitating creative processes and artistic outcomes. To that end, the next section considers examples of artists working creatively and collaboratively with robots.

### 2.3 Robots and Art

The impact of computers on art making is well-established, but only recently have researchers begun to seriously consider the role of robots in art making. Given the connections between computer art and robotic art, it is surprising how little overlap there is in scholarship that addresses their entangled histories. Our interest in drawing robots is motivated by a broader interest in exploring how the performing and visual arts can open up new pathways for robotics and embodied interaction (Jochum et al., 2017; Jochum and Derks, 2019). Following Madeline Gannon’s work, we recognize how robots act as bridges between virtual and physical worlds (“their minds are in the virtual, but the bodies are in the physical”) and as such they are not necessarily well configured or equipped for reacting to changing environments or open-ended control (Gannon, 2018, p.138). A robot’s physical embodiment and material instantiation give rise to a particular set of concerns that computer art does not; embodiment has practical implications for how robots perceive and navigate the world and also for how we design systems to control and operate robots (Fdili Alaoui et al., 2015) (Wainer et al., 2006). We expand on the discussion of embodiment and interaction in Section 6. Many pioneering experiments in art and engineering collaborations are collected in (Salter, 2010), which includes examples from early art and technology performances (Loie Fuller’s work with dance, film and lighting) and pioneering robot art works by Bill Vorn and Louis Philippe Demers (Vorn, 2016) (Demers, 2016). Within the field of robotics, Amy Laviers (Ladenheim et al., 2020), Catie Cuan (Cuan, 2021), Petra Gemeinboeck (Gemeinboeck, 2021), and Marco Donnarumma (Donnarumma, 2020) have experimented with research strategies that explore dance and other forms of corporeal expression between human and nonhuman performers. While these works vary widely in aesthetics and approach, they all share a commitment to exploring the entanglement of human-machine interaction through the staging of imaginative embodiments. Donnarumma uses the term “configuration” to denote the “performative assembly of human and nonhuman parts to create alternate forms of embodiment” (Donnarumma, 2020, p.37). These are only a handful of examples of trandisciplinary research investigations that allow artists and engineers to explore creative processes together towards new outcomes and insights. 

Closer to the domain of visual arts, there are several examples of sophisticated robots drawing systems that generate drawings and dexterously work with physical materials to produce impressive drawings and paintings, including (Gülzow et al., 2018; Still and d’Inverno, 2019; Smith and Fol Leymarie, 2017; Berio et al., 2016; Santos et al., 2020). In these instances, the collaboration between artist and tools for the most part happens *via* the software, and the human artist’s physical interaction with the robot is not in focus. Other artists choose to work more directly with the artist-tool-material frame, combining human artists with robot tools in real-time interaction in shared physical spaces. Sougwen Chun’s collaborative drawing performances Chung (2015) and Patrick Tresset’s interactive portrait drawing robot (Tresset and Deussen, 2014) are two examples of drawing robots that account for tools as technical ensembles and explore new art making practices between humans and machines. Chun’s drawing performances with D.O.U.G. (Drawing Operations Unit Generation 1) began with simple mimicking gestures (similar to the pantograph), where a small robot arm reproduced Chun’s physical gestures in real-time on a shared canvas. It was the exploration of the materials, especially the unintentional marks that punctured or slipped on the canvas, and Chun’s improvised responses to these spontaneous and unplanned actions that render the work compelling for the artist. Similarly, Tresset’s performance installations with RNP, a custom robot art and computer program for real-time portrait drawing, interrogate the role of physical presence and embodiment. The robot is programmed to draw in the artist’s individual style, but the tools and materiality of the system (ballpoint pen, canvas, writing desk, robot arm controlled by servo motors, the webcam that observes the sitter and performs small animations) all direct attention to the larger socio-technical context in which the art work occurs. In both works, audiences do not merely observe a robot that makes art but are invited to observe the creative process of the technical ensemble at work, watching how human artists and robot tools continually modify and shape one another. Bruno Latour famously observed that tools are “the extension of social skills to non-humans,” and these performances poetically explore the implications of tools that exhibit social and artistic agency (Casper and Latour, 2000). These art works propose different models of interaction in robotic art that account for the dynamic and temporal aspects of drawing and evidence how artists and machines can work collaboratively and creatively in ways that are not predetermined. We hope that a discussion focused on processes of becoming and collaborative creativity between artists and machines can help avoid dualistic thinking of creativity as an either/or proposition (either a machine can be creative or it cannot). We are less interested in replicating the artist’s process than developing a better understanding of how robots can meaningfully participate or intervene in creative processes and designing tools that support such participation.

## 3 Methods

The diverse methods used in this study reflect the transdisciplinary nature of the research team. We draw equally from the fields of arts and humanities, engineering, computer science, and human robot interaction research (HRI).

### 3.1 Artist-in-the-Lab/Researchers-in-the-Atelier

Some models of creativity consider creativity to be an internal and solitary process, while others view creative processes as collaborative, improvisatory, and social. We initiated a series of workshops within the artist-in-the-lab framework. While every member of the research team has some level of artistic background, the named artist on the project, Valeria Rizzo (Rizzo), was hired to work alongside academic staff. Other members of the research team came from diverse backgrounds: Carlos Gomez (Gomez) is formally the project engineer and also a musician; Maros Pekarik (Pekarik) is a creative technologist working with interactive media and projections in live performance installations; and Elizabeth Jochum (Jochum) is a human-robot interaction researcher with formal training in theatre, dance and puppetry. The project was assisted by Andreas Kornmaaler Hansen, a graduate student in Engineering Psychology. The workshops alternated between university laboratory facilities and the artist’s studio. The workshops were characterized by an exploratory, generative view of art making. The collaborative nature of our investigation acknowledges the significant role that peers play in creativity (Csikszentmihalyi, 1998). In this case, peers were not just the social environment or judges but included other lab members involved in a related research project (Jochum et al., 2020). Too often, we observe that artists are invited into research labs as creative provacateurs or instigators but rarely participate as full members of the research team. Our collaboration revealed the very concrete institutional obstacles when hiring artists to do research. It also revealed the challenges of working across disciplinary borders, especially when working with technologies that require specific knowledge or competencies (for example, programming robots). It is worth noting that these challenges are not rendered visible when artistic outcomes are presented at festivals or museums; nor are they traditionally discussed in literature. Despite the institutional and conceptual challenges of trandisciplinary research, the possibility of sharing material with multiple creative agents (e.g., other researchers) from various domains allows for more complex re-interpretations of the material. We wanted to create a rich environment across different conceptual spaces where all members of the research team could participate and contribute equally. Therefore, the project involved close, sustained collaboration where the researchers met regularly over the course of several months. The frequent exposure to other methods of working presented opportunities to participate meaningfully and learn from one another.

### 3.2 Workshops

The first workshop was conducted in Rizzo’s studio, investigating aspects of collaboration through collaborative drawing and painting techniques and tools. We explored how these techniques could be adapted to the context of robot-human collaboration and attempted to better understand the artist’s creative processes. The second workshop took place in a robotics lab, with an emphasis on trying out new techniques with the robot and observing the interaction between Rizzo and the robot. Alternating between these two workshop formats and locations, we explored working in two specific domains of creative collaboration. The primary aim was to use all the tools with similar capacity so the artist did not have to rely on engineers to get things working, and the engineers did not rely on the artist for specific instructions.

### 3.3 Video Cue Recall

Video cue recall (VCR) is an ethnographic method used in the social sciences and humanities (Bentley et al., 2005). Originally intended to help reduce bias in self-reporting protocols, this qualitative method aims to elicit concrete feedback from participants regarding their experiences or to conduct domain analysis. VCR has also been used in human-centered computing to gain insights into interaction behavior. We replayed the video of the entire performance of If/Then (2020) in the presence of all authors. First, Rizzo was asked to comment on her overall reactions to the performance. Then Gomez, Pekarik, and Jochum took turns posing questions and asked Rizzo to comment on specific moments in the performance. The session was recorded and transcribed using SonixAI automated transcription and reviewed and corrected by Jochum, Gomez, Rizzo and Pekarik. Jochum then reviewed the transcripts and the authors coded them according to thematic analysis. All first-person quotes from the research team that appear in this paper were obtained in this manner.

## 4 Materials

Collaborative robots, also known as cobots, are a special class of machines. Cobots are an increasingly significant branch of industrial robots with a particular advantage over other types of industrial robots: they are designed to work in close proximity with people and are equipped with security features that adjust the force and speed of the movements to render them safe for close interaction.

### 4.1 UR3

The main hardware is the UR3, a cobot manufactured by Universal Robots. It is the smallest of the series, with 6° of freedom and a reach of 50 cm. This high precision robot is able to move at high speed while maintaining high levels of accuracy.

### 4.2 Initial Software

The initial drawing software was developed by Hinwood et al. (2018) and described in (Hinwood et al., 2018). The software was initially developed as a tool to study human robot interaction during a collaborative drawing task (Pedersen et al., 2020). The program works as follows: first one calibrates the real world coordinates of the canvas. Then raw images are entered as inputs to the software where the contours of the objects are extracted into key points, which are then translated to real-world coordinates. These coordinates are sent to the robot sequentially, so the program plots the contours that result in a drawing. The software also allows one to store animations by manually introducing a few select robot poses that the robot can execute sequentially to make the robot appear expressive and communicate with the drawer. The program uses a blocking protocol to interface with the robot, which ensures controlled speed and acceleration and making the robot safe to interact with. One drawback of this system is that the interface is blocked until each command is finished, meaning there is no possibility for real-time control. Our experience with Section 4.2 informed the development of the new software program EMCAR Section 4.3.

### 4.3 EMCAR

**FIGURE 1:**
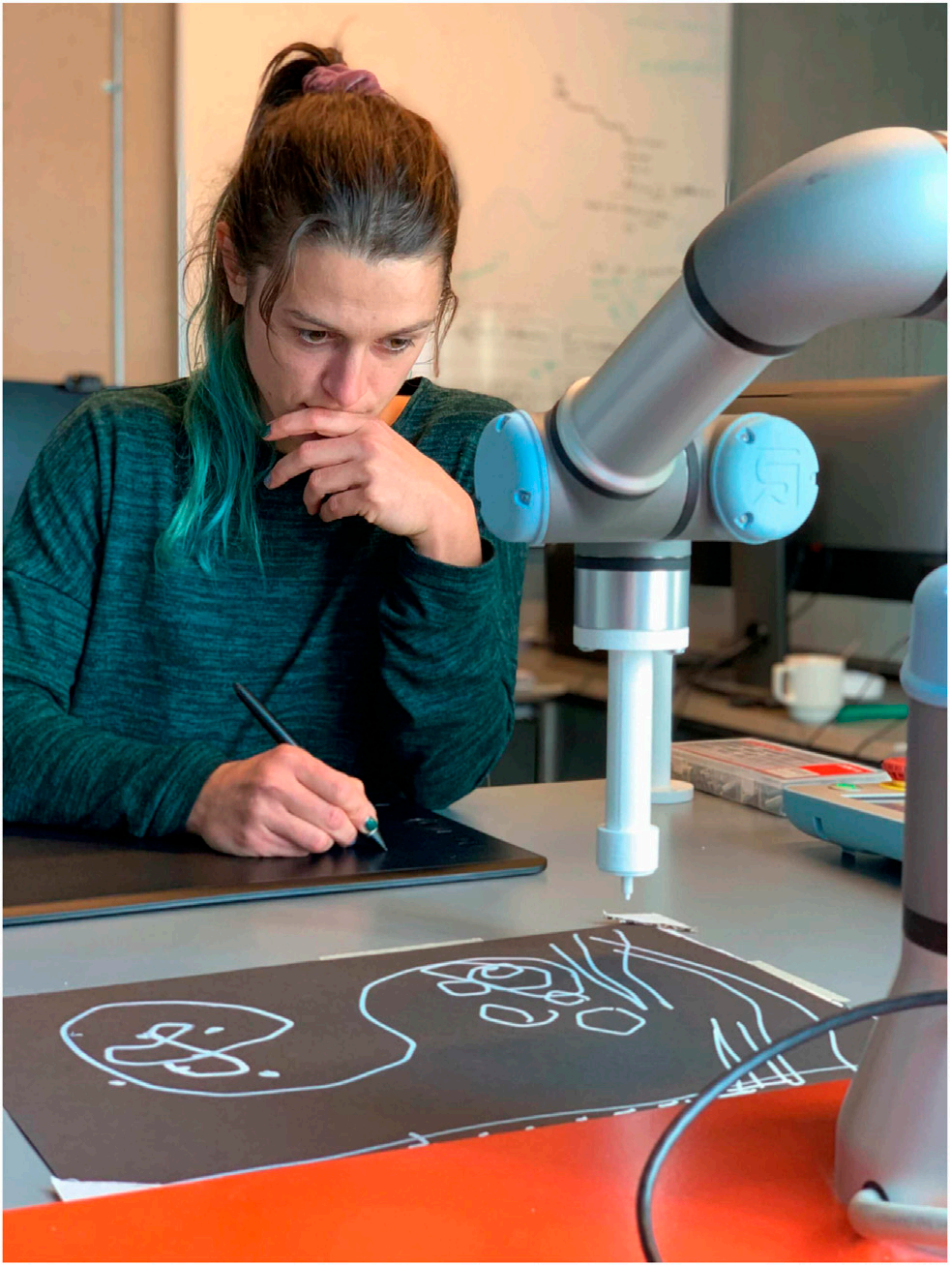
Rizzo in the HRI lab, experimenting for the first time with teleoperating the UR3 using a stylus and digital tablet. The program had several modes: mirror, follow, and replay.

To overcome the limitations of the early system, we developed the Embodied Controller for Animating Robots (EMCAR), a custom software tool for controlling an industrial robot arm that offers direct, embodied interaction for generating and programming animation sequences by manipulating the robot freely. This technique gives people with little technical knowledge the opportunity to work directly and intuitively with the robot as they would with other materials. EMCAR makes generating robot performances easier by allowing people to directly puppeteer the robot, and also allowing for real-time teleoperation by using different inputs. For example, a Wacom digital drawing tablet allowed a person to control the motion of the robot directly through a stylus, shown in Figure 1. The EMCAR implementation is built on top of open-source software and made available to the community^2^. EMCAR was subsequently stress tested in the development of a human-robot dance performance described in Section 5.2, where a dancer interacts with a robot in two modes: one as a puppet with pre-recorded movements and the second with her body, making use of a depth camera and computer vision software that maps the dancer’s body position into the robot task space. Although it was developed as a tool for artistic performance, EMCAR has potential for diverse applications beyond art.

#### 4.3.1 Real Time Robot Interfacing

Interfacing with a robot typically involves using ROS (Robot Operating System) or the robot’s individual API, using functions that block the robot until it performs a certain action. This means that the robot can be easily tele-operated, with the limitation that the robot is prevented from doing any other movement until the command is completed. This feature gives full control of different parameters, such as speed or acceleration, while ensuring millimeter precision. On the other hand, it means the robot cannot adapt to rapid changes and fluid inputs as would be expected in a performance. In other words, the system is not Real-Time Controllable. RTDE, which stands for Real Time Data Exchange, is a protocol recently implemented by Universal Robots which allows to interface the robot in real-time. The robot runs a loop with a short time of iteration from 0.4 to 2.0 s. At the same time, an external device can stream to the robot a target position, which is updated several times per second. At the end of every iteration of the robot loop, it will try to reach the last target position read. This leaves the robot free to adjust the speed and acceleration to meet the target position in time, which can be dangerous in close interactions with people and objects. Using a short iteration time (i.e., 0.8 s), it can smoothly follow any trajectory generated in real time which doesn’t contain abrupt changes during the iteration time. It also introduces an intrinsic latency of this time, which is noticeable when tele-operating the robot and compromises the detail of the drawing in favor of performance time.

#### 4.3.2 Multimodal Robot Interfacing

**FIGURE 2:**
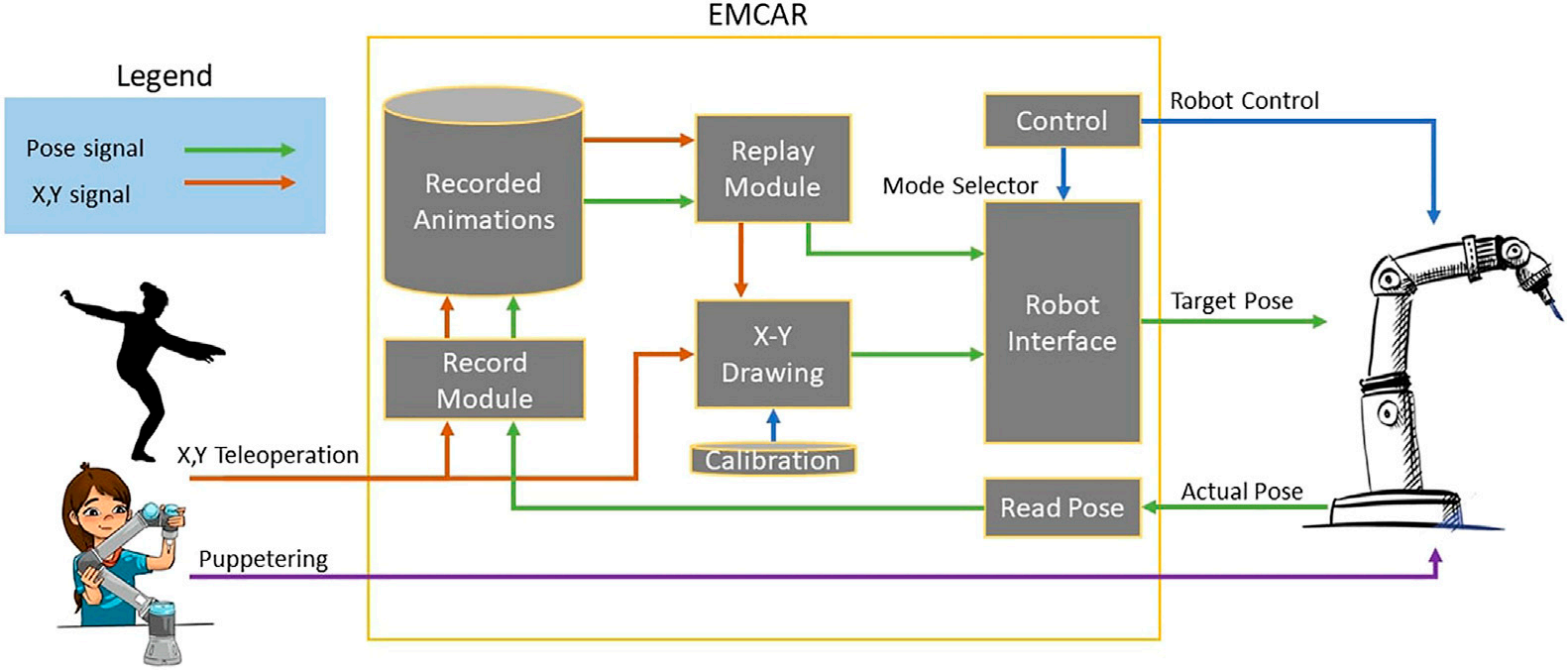
Diagram of internal functions of EMCAR.In the left are the different inputs, X-Y position and Puppeteering. In the right is the robot interface. In the middle, inside of the orange square is the internal modules of EMCAR that create its functionalities.

As a result of the different workshops described in Section 3, two different modes of interfacing with the robot were designed: X-Y Painting and Puppeteering. All this was commanded using a simple graphic user interface that allows access to all the functionalities with a simple mouse click. The diagram of the internal workflow of EMCAR is described in Figure 2 and is explained in the following paragraphs. For the X-Y Painting mode, we designed an intuitive calibration process that allows the artist to point the four corners of a canvas on a horizontal table. This process stores the real-world coordinates where the canvas is located. After calibration, EMCAR can receive X-Y coordinates from an external device or software (for example a stylus or another sensor) and map this position into real world coordinates of the canvas and send them to the robot. As a result, the artist can use, for instance, a drawing tablet to draw together with the robot. As the X-Y input is agnostic, any other input in this format is valid. During the performance of *If/Then* the input was the X-Y coordinates of the artist on the dance floor, extracted using a depth camera with computer vision, so the robot could be controlled through the artist’s movements. 

For the puppeteering mode, another approach was used. Here the robot is set to “free mode,” which releases the motors and allows the artist to adjust the robot manually into a desired pose. Position information for each pose during the sequence is retrieved in samples by EMCAR and stored. Each sample is composed by a time stamp and the endpoint of the end-effector. While recording, the artist can puppeteer the robot in an intuitive manner with instant feedback of what the animation will look like. This technique provides a sensuous and tactile experience for the human artist, allowing her to explore the textility of making, as a puppeteer might. The process is redolent of Ingold’s description of how an artist does not impose form, but rather learns to “follow the forces and flows of materials that bring the form of the work into being” (Ingold, 2009, p.97). The embodied controller has an added advantage in that it saves an enormous amount of time and creates lifelike movements that cannot be achieved as easily with other animation methods. Once the animation is recorded, it can be replayed: EMCAR sends the desired poses in real-time, using the same time of iteration between samples used during recording. We elaborate on the implications and assumptions of embodied interaction in Section 6.

#### 4.3.3 Recording and Playback

Following the puppeteering approach, X-Y Painting was also developed to record and save a drawing as an animation. As a result, the artist can store new drawings and puppetry animations in different animation banks to be used later when devising performances. These animations can be replayed by the artist (or a second operator) during a performance, moving between tele-operation on the fly and pre-recorded animations, giving more flexibility to scripted performances using both techniques.

### 4.4 Tablet

Graphic drawing tablets are widely used devices. They consist of a sensitive surface and a special pen that digitizes the physical strokes of a person drawing. The simplest dataset that can be obtained is the real time X-Y position of the pen, which includes information about whether the pen is touching or hovering over the tablet, and the applied pressure and angle of approach. The tablet provided a straightforward and embodied method to control the robot and produce new drawings. At the same time, it is an excellent tool for instantly obtaining X-Y positions, and was used extensively during development and troubleshooting. It quickly simulated any X-Y position generator; for example, in the performance the X-Y position was retrieved from a depth camera using motion tracking software.

### 4.5 End Effectors

**FIGURE 3:**
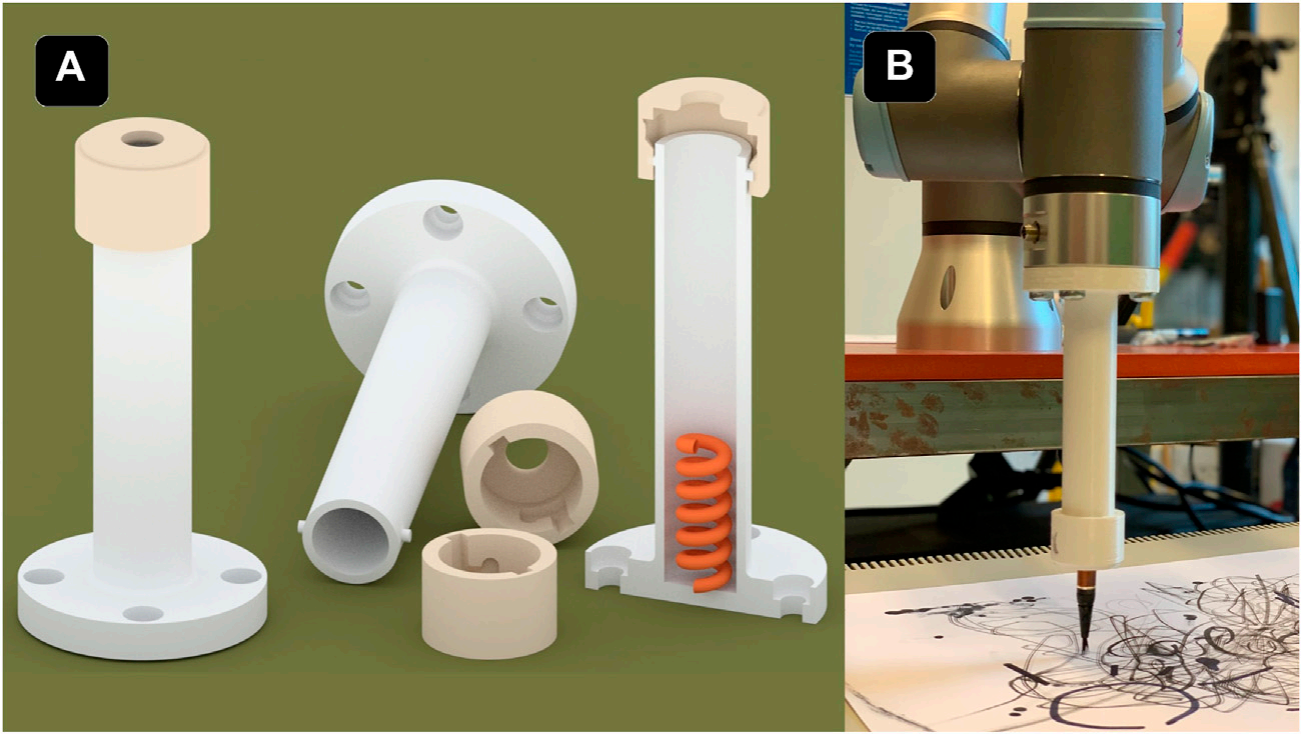
**(A)** 3D representation of three of the objects used as an end effector to attach different drawing tools, such as markers or brushes. It contains a sprint in the bottom to adjust the tool. The object at the right is cut in half to display the inside.**(B)** Image of the end effector with ink brush attached to the UR3.

The end effector used for drawing is a 3D printed tool, shown in Figure 3, that allows us to attach different drawing tools (e.g., pens, markers and brushes) to the robot and thereby expand the artistic possibilities by allowing for a range of drawing and painting instruments. It consists of a hollow cylinder with a spring in the bottom and a cap that can be adjusted with a notch. The cap has some millimeters of headroom, allowing a small tolerance that allows for some give when drawing. The main drawing instrument used during the workshops were a ball pen with a ultra thin trace, different common white board markers with a thicker trace, a Chinese ink brush that plotted a more organic-looking stroke, and finally a professional thin white marker over a black paper that created more striking contrast and had a better finish. The strokes were unique to each instrument, and we experimented with different tools (ink brushes, pencils, markers, etc.) that each altered the appearances of the drawings. For *If/Then*, we chose to use the white marker on a black canvas, as it generates more aesthetically appealing results in a dark performance space. Markers and ball pens were used primarily in the workshops and development because of their robustness and low cost.

### 4.6 Projections

During workshops, we experimented with floor projections. Two ultra short throw projectors were mapped and aligned to create an interactive display on the floor. The projectors were positioned facing each other to create a seamless image by eliminating shadows. A depth camera made it possible to track the position of the performer in the space using simple computer vision techniques such as background subtraction and blob detection. The field of view of the camera was mapped with the range of the projectors that allowed for the ability to project objects at the artist’s feet according to either the robot’s position on the canvas, or the artist’s position in the room. Projection mapping and computer vision processing were made in the TouchDesigner (Derivative, 2021), a node-based programming language for real-time interactive multimedia applications.

### 4.7 Limitations

It is important to acknowledge the specific limitations of both hardware and software in our project. The main limitation we experienced was the robot loop time, mentioned in Section 4.3.1. The RTDE protocol has an intrinsic robot loop time, where the robot tries to reach the position sent in the previous iteration. This time lasts 0.8 s, which introduces a corresponding delay. Most importantly, it overrides the data that is received between iterations, meaning that positions with less than 0.8 s are lost, introducing a “low pass filter” of the drawing strokes. Therefore, if the artist draws a zig-zag line with a frequency higher than 0.8 s, the robot won’t be able to draw in time. This limitation also applies for the puppeteering function, but is less noticeable because significant changes occurring in less than 0.8 s only occur when moving the robot in a very aggressive manner.

## 5 Results

Identifying which outcomes qualify as results can be difficult—and perhaps even paradoxical—when reporting on process-led (as opposed to product-driven) creative practice. To narrow our focus, we include a summary of the workshops wherein we identified specific artistic processes of visual art making. We then report on some of the other tangible outcomes, including a live dance performance. We also include results from the video cue recall session we conducted with the research team, as this yielded insights relevant to our discussion.

### 5.1 Workshop Summary

**FIGURE 4:**
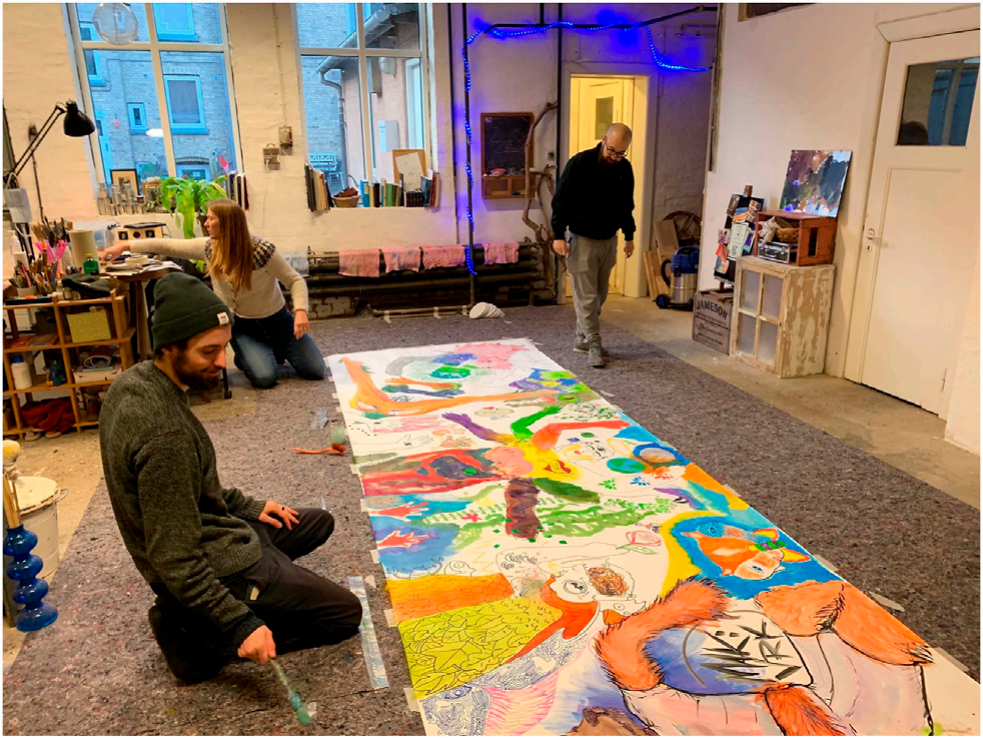
The research team engaged in weekly workshops at the artist's studio, engaging in collaborative drawing and painting tasks, including collaborating on a large physical canvas.

**FIGURE 5:**
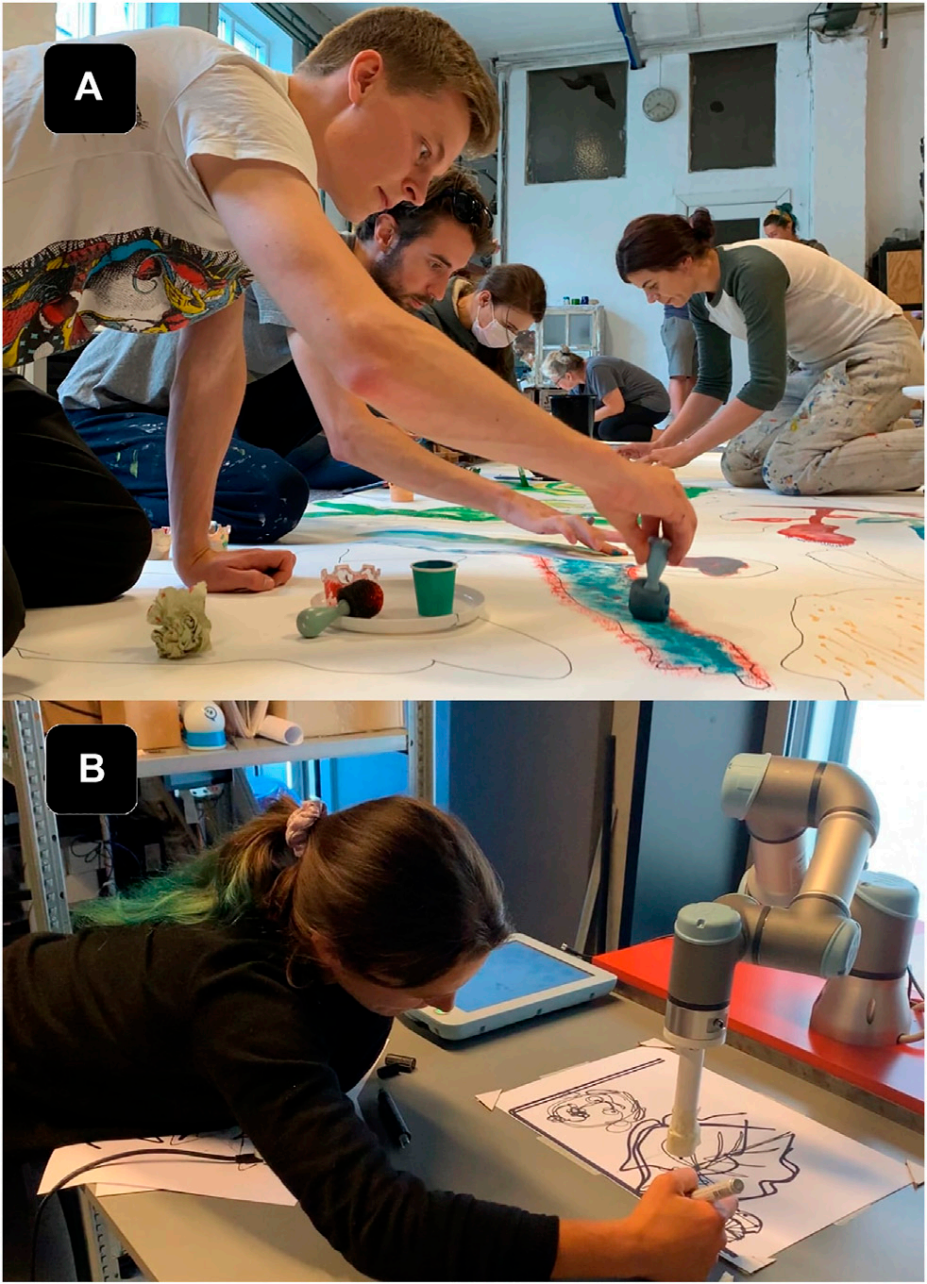
**(A)**The research team engaged in several workshops at the artists' studio, exploring various painting tools and techniques with different instruments (sponges, stencils, ink pens, pipettes, etc.). **(B)** Artist (Rizzo) at the HRI lab, testing an initial drawing collaboration using EMCAR with the UR3. EMCAR allows the artist to draw simultaneously on a shared canvas in real time, as in the artist's studio. The system was an improvement over the pre-programmed drawings of the previous version of the software.

As mentioned in Section 3.2, two types of workshops formed the core of our research investigation. Both workshop formats were inherently distinct and designed to move the research team out of our comfort zones while also allowing space for knowledge translation between the fields. Initial workshops aimed to be a place for exchanging perspectives by introducing respective processes to one another. Rizzo led workshops on collaborative drawing and painting in her atelier. During these participatory workshops, the research team was invited to work together on a shared canvas (Figure 4) and to experiment with different materials and tools that are part of the visual artist’s toolbox. The team spent time with various tools, trying to understand the internal process of what might encompass a drawing experience through physical interaction with the materials. The second series of workshops were conducted in the HRI lab at the university, where the research team tried to identify and translate the knowledge gained from the collaborative drawing workshops to specific methods of collaborating and co-creating with the robot (Figure 5). Gomez and Pekarik demonstrated UR3 capabilities through an existing software for collaborative drawing described in Section 4.2. As the software only reproduces pre-made drawings, the interaction was simply too narrow for Rizzo to work with. Thus, we decided to extend the design requirements. Observing and experiencing first-hand how human collaboration and co-creation developed during the workshops in Rizzo’s atelier, the team understood that the real-time human’s creative contribution as an input to the system and applicable responsive output from the robot might open more possibilities for creative encounters. Therefore, we prioritized the development of a system capable of real-time robot interfacing, eventually called the EMCAR tool described in Section 4.3.

**FIGURE 6:**
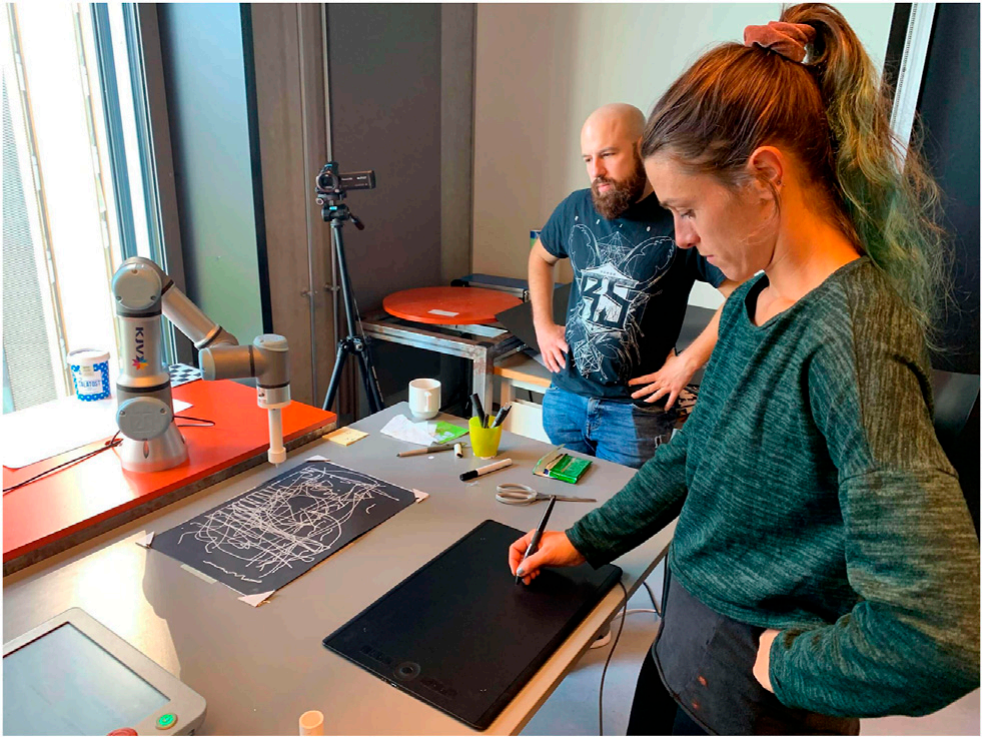
The research team in a workshop at the HRI lab, experimenting with an early prototype of teleoperating the UR3 using a stylus and digital tablet.

The combination of artists’ backgrounds in dance, circus performance, theatre, puppetry, music and interactive art influenced the next development stage. Consequently, we expanded the activity outside the drawing format, which led us to devise a performance for live audiences. We implemented tele-operation features controlled with the stylus (described in Section 4.3.2 and seen in Figure 6), which gave Rizzo a sense of the ability to control the robot and produce drawings. However, the full control over the system had adverse effects on the aspects of co-creation. Rizzo was less interested in having precise control over the robot to intentionally make marks on the page, and more interested in interfacing with the robot intuitively, the way she worked with other tools and other artists in the first workshops. What is important to note is the transition from visual art to the study of physical motion. The experiences of co-creation between human-partners through tools on a shared canvas opened up a line of inquiry that we had not fully considered: the movement of the artist and the negotiation between the human artist and the robot was essential for creating an experience of collaboration. From the second workshops, Rizzo expressed an interest in moving together with the robot to produce a drawing, and thus our focus shifted to creating an interface that allowed Rizzo to work in a more physical, though less deterministic, way with the system. We focused on ways to translate Rizzo’s motions to the robot’s body, drawing on corporeal empathy and making explicit the connection between human-robot-tool (Fdili Alaoui et al., 2015; Sheets-Johnstone, 2011).

**FIGURE 7:**
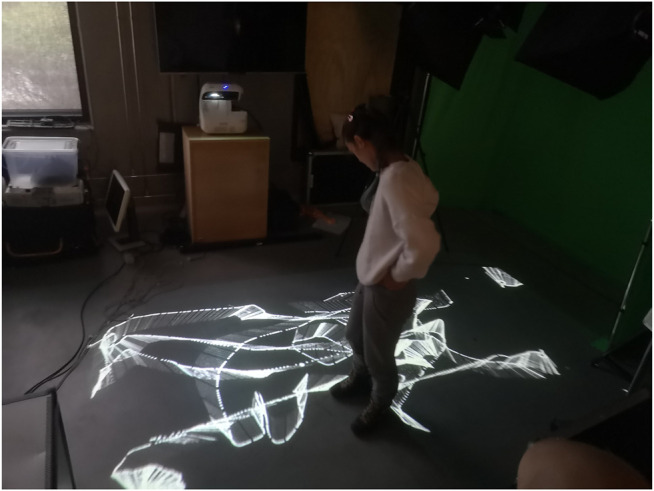
Interactive projections on the floor track the motion of the Movement trajectory traces represented with a projection on the ground.

Once we translated dance moves to the robot’s motion, we began to conceptualize the entire physical space as a canvas. The artist’s position and movement in the space were highlighted by projected animations on the floor described in Section 5.2.3 and seen in Figure 7, mirroring the stroke on the physical canvas drawn by the robot. It was important to work with a technology that would allow Rizzo to have freedom of movement without any unencumbered body movement. Pekarik and Rizzo had previously collaborated on an experimental performance involving physiological sensors, motion tracking and dynamic projection mapping^3^, and Jochum had previously developed an improvised robot-dance performance (Jochum and Derks, 2019). Given our shared background in performance technologies, we decided to work with the combination of depth camera and projection mapping techniques to make the material more tangible for the performers and the audience.

**FIGURE 8:**
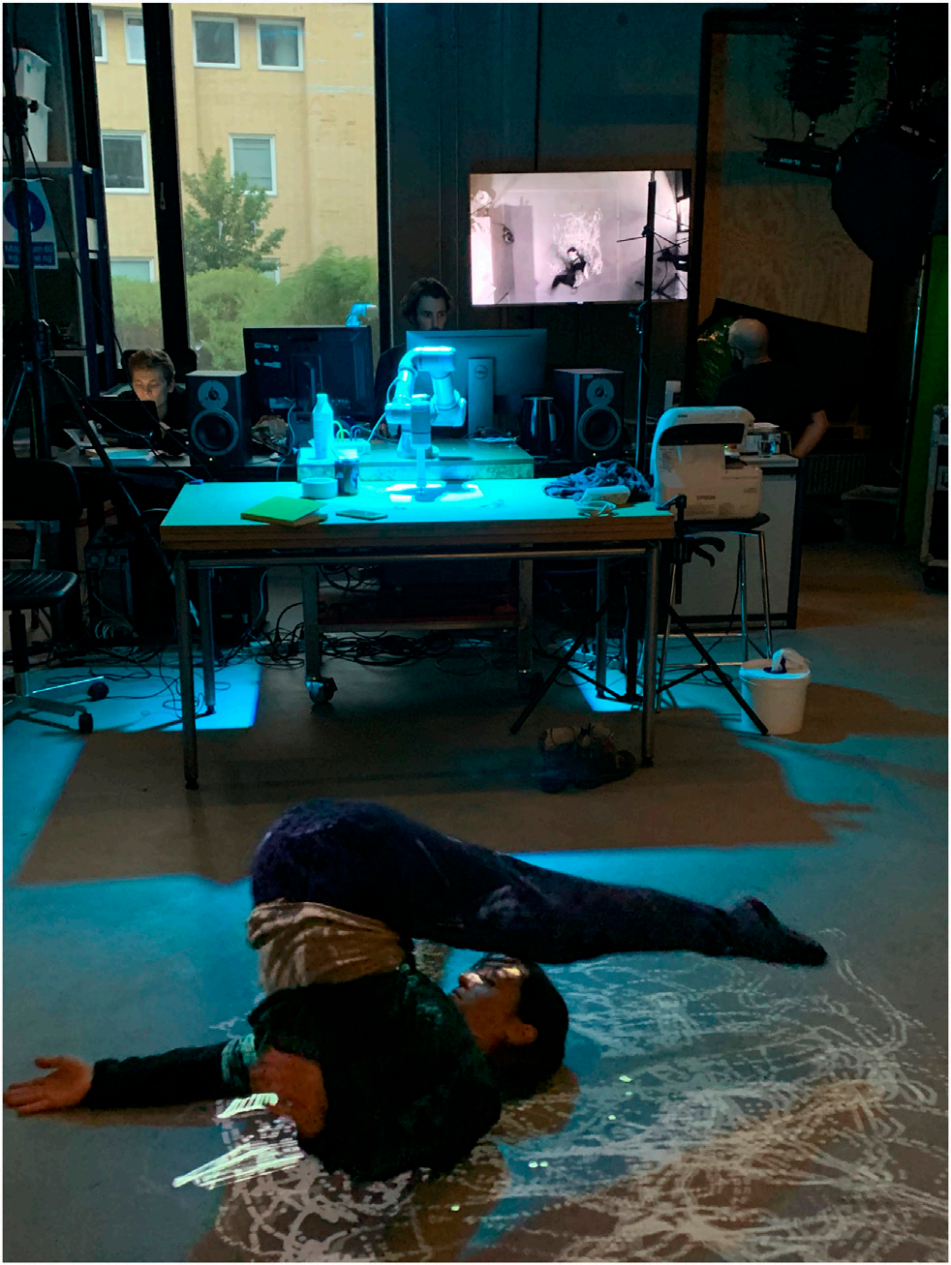
The research team iterating the design for the projection animations and performer tracking during a workshop at the RELATE lab. On the screen is projected a real-time feed of the performer and animations captured from a video camera mounted above.

**FIGURE 9:**
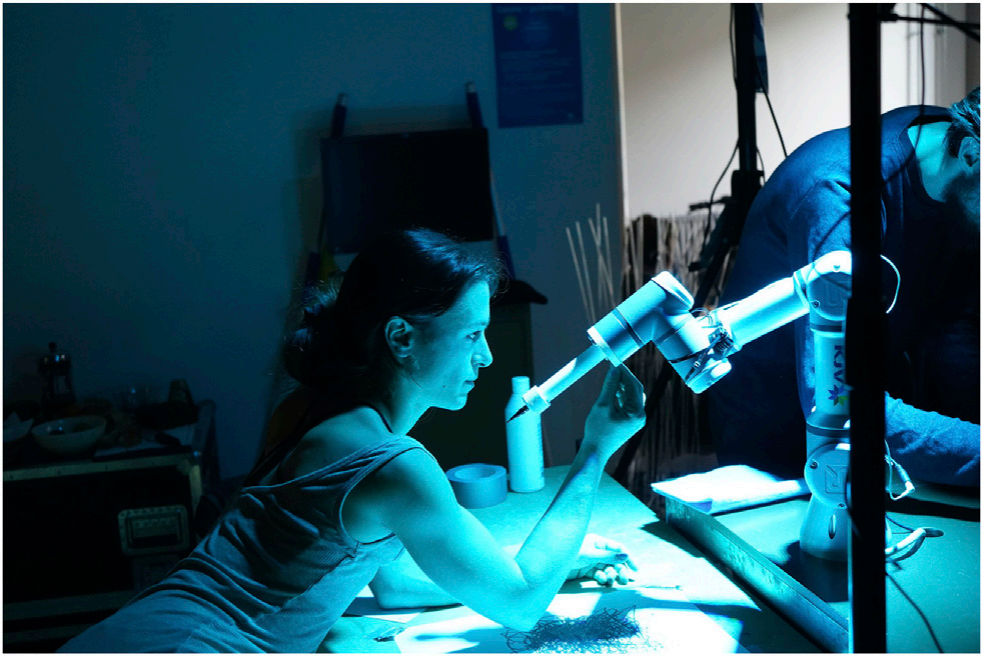
Rizzo works with the robot during a puppetry workshop at the RELATE lab. Using physical manipulation, EMCAR allowed the artist to work directly with the robotic arm to choreograph, record and playback animation sequences.

The last workshops in our process focused mainly on devising the performance of *If/Then*, incorporating sound, lights, live-feed video cameras, and visuals that complement the performer’s actions, shown in Figure 8. The choices concerning the narrative are presented in Section 5.2.1. To further convey the narrative aspects of the performance, the EMCAR tool was extended with a puppeteering mode described in Section 4.3.2. The immediate recording and replaying of the animations offered a high level of physicality and embodied interaction which allowed the team to work together to intuitively explore possible motions, illustrated in Figure 9.

### 5.2 Performance

**FIGURE 10:**
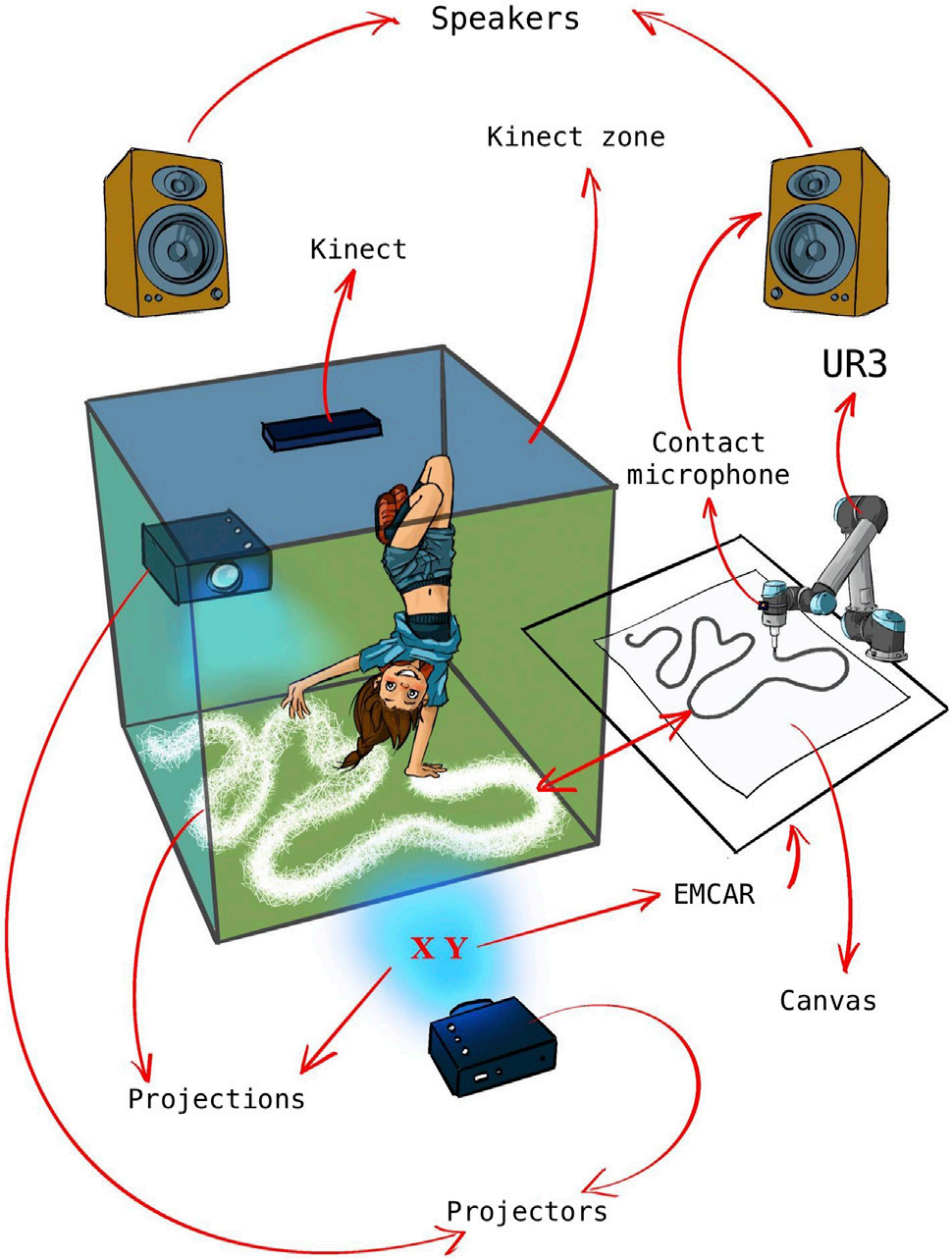
Overview of the final of the system and implementation details for the live performance. A projector mounted on the ceiling projected real-time video capture from the performance from four different angels (including a camera mounted directly on the robot) on one wall in the gallery space. Two floor mounted projectors using sensor data from the cameras and projected lines that corresponded to the movement patterns of the dancer with lines on the floor.

The outcome of the workshops resulted in an original performance staged three times at the Danish National Museum of Science and Technology. The performance was conceived as a complementary program for the interactive drawing installation that ran during the day. The performance was not meant to be a final showing, but rather a public demonstration of a work-in-progress. The duration was around 15 min, and was performed on the half hour, with three performances in all.^4^ In Figure 10 is presented the setup used in the performance. The initial agreement with the museum was to exhibit an interactive drawing robot, similar to the one described in (Pedersen et al., 2020), although this time with a smaller robot (UR3 instead of UR10). The process of generating a public performance in a space that was not designed for performance, and with a producing partner with no prior experience with live performance events, meant that the performance came into being because of circumstances surrounding the installation rather than a specific idea or pre-formulated script. In this way, it echoes the process-oriented view of artmaking and technical ensembles view described above. The narrative and dramaturgy of the performance was a direct outcome of the practical necessity of running an installation during the day that would seamlessly transition into a performance without disrupting the museum. The public presentation, although documented and recorded, was never considered a finished product, but a material expression of the investigation of the limits and possibilities of real-time human-machine interaction, a process of becoming as described by Ingold.

#### 5.2.1 Narrative

**FIGURE 11:**
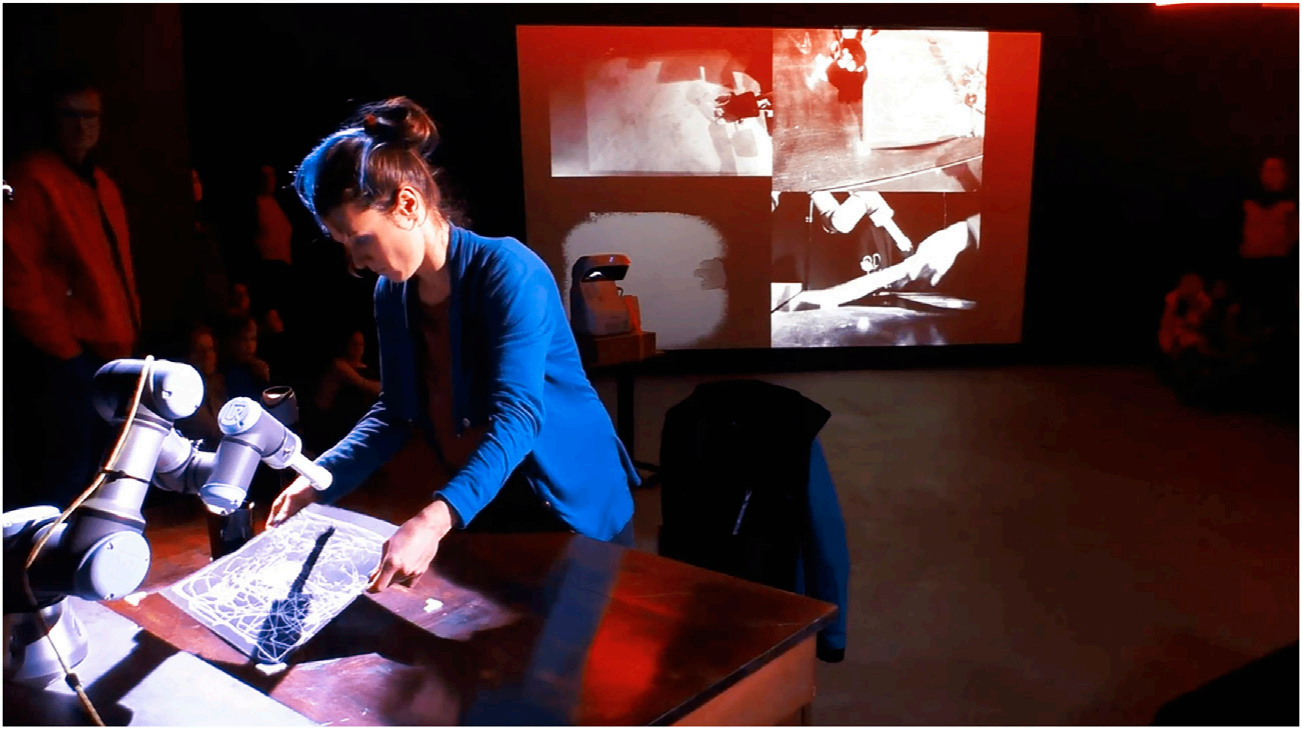
The team performs *If/Then* performance at the museum. Rizzo works with the robot and executing a choreographed sequence in front of the audience. She controls the robot in “free mode” to get a drawing out of the drawing area.

The ability to experiment physically with the robot and work with the projection system inspired Pekarik and Rizzo to form a narrative around the already existing research activity conducted with the robot. Therefore, Rizzo’s role as a research assistant was integrated into the final narrative in favour of our research continuation to explore her partnership with the robot further. The narrative followed the journey of a team of researchers undertaking a routine examination of a robotic arm tasked with a routine drawing operation (standard procedure during our investigative practice). Arriving at the workplace, the researchers find the robot stuck with an unexpected drawing output on the desk, shown in Figure 11. The anomaly indicates a possible bug in the robot’s system that could have occurred overnight. The operators (members of the research team) restart the system and perform check-ups that confirm the robot is ready to resume work. However, during these procedures, the robot becomes distracted and breaks away from the task to look around. The researcher tasked with supervising the robot suddenly notices the strange behavior, and the robot ceases the predefined drawing task and begins to create a new drawing that is mapped to the researcher’s position in space. Taken by surprise, the researcher responds with curiosity, and subsequently engages in a movement exploration to investigate the mappings of her motions in space to the robot’s movement on the canvas, and the corresponding light drawings that are projected in real time on the floor. The exploration intensifies until the robot suddenly drifts off the canvas, inadvertently scattering items across the table. The performance ends with researcher and robot facing one another in tableaux.

#### 5.2.2 Puppetry

**FIGURE 12:**
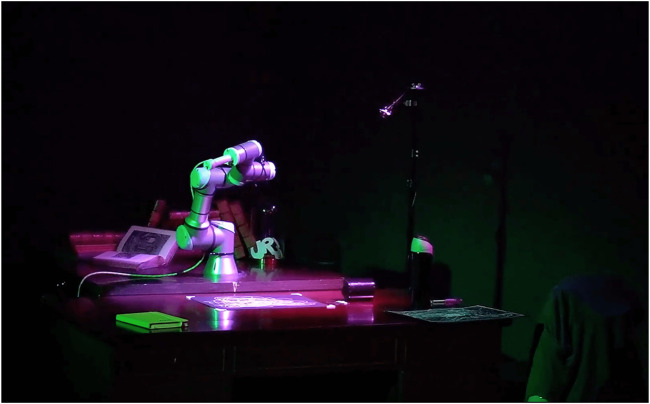
The robot after performing animation of hitting a cup from the table during the *If/Then* performance at the museum. EMCAR was used to record the animations before the performance and replay them in the cue moments.

During the workshops, the performer recorded various robot animations with EMCAR’s recording functionality described in Section 4.3.3. Two types of animations were recorded, using puppeteering mode and drawing mode described in Section 4.3.2. In puppeteering mode, the artist recorded animations by physically manipulating the robot to the desired sequence. This made it possible to create expressive human-like animations for the robot like the “hitting the cup” shown in Figure 12. The drawing mode allowed the artist to recreate a real-time drawing process captured on the tablet, and was used for the drawing loops on the canvas. No attempt was made to hide or mask the operation of the robot: during the performance, animations were cued and executed by team members seated onstage and in full view of the audience. The performance combined a mix of pre-recorded and live tele-operated actions that, together with the performer’s improvisations, meant a unique performance (and drawing) each time.

#### 5.2.3 Interactive Projections

**FIGURE 13:**
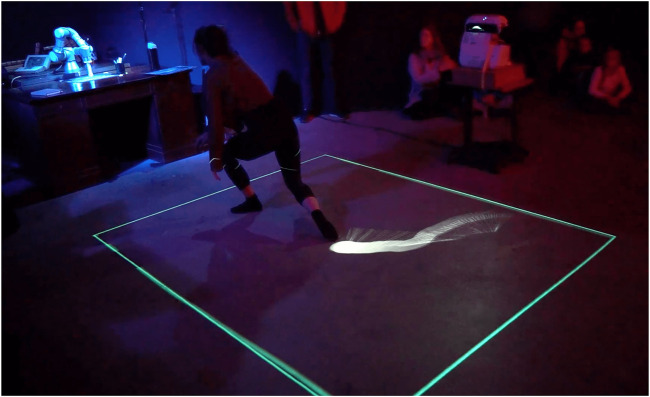
Projectors facing each-other, creating an interactive display. The display is mapped with the tracking camera and the robot canvas. The floor becomes visual feedback of the artist’s movements controlling the robot.

**FIGURE 14:**
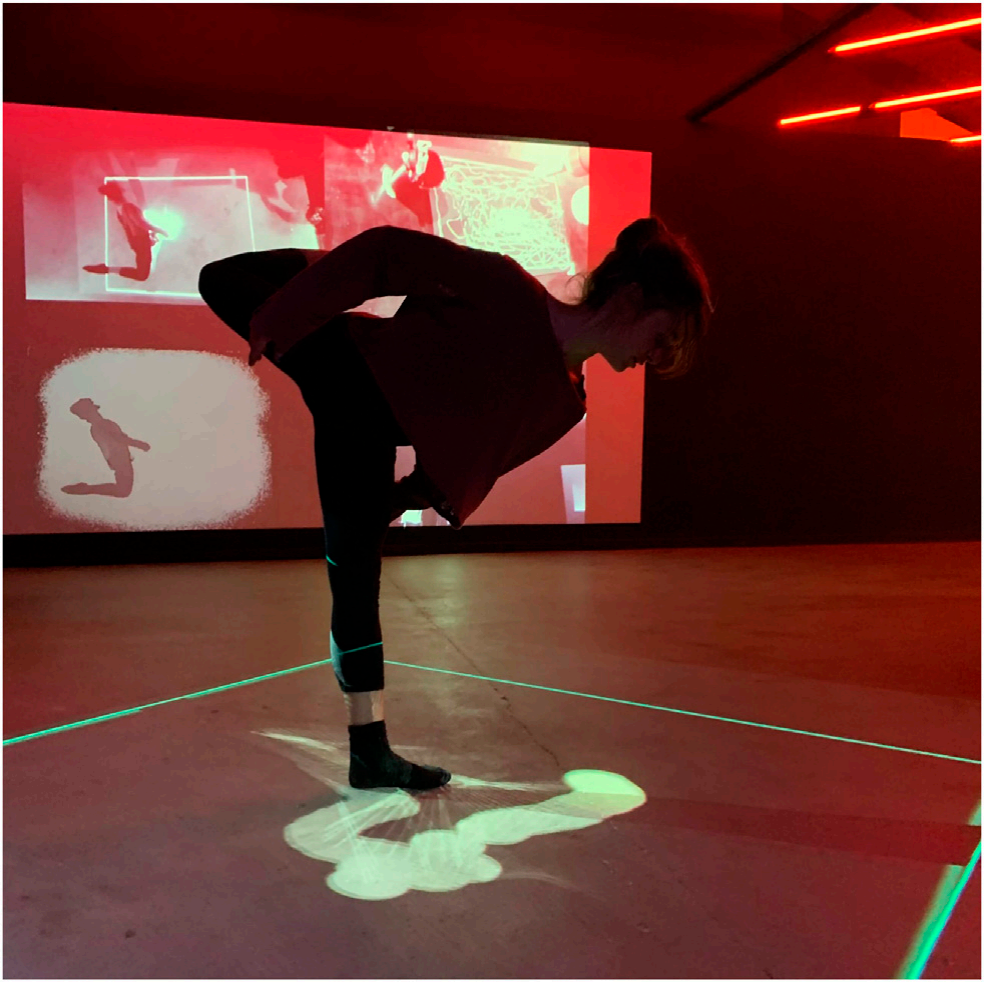
The artist performs *If/Then* performance at the museum. The four channel projections on one wall of the gallery were comprised of four real time cameras that alternated throughout, giving the audience a chance to observe the performance from various perspectives. The video channels were VJ’ed live by Pekarik. Jochum designed and operated the lights, and Gomez performed live mixing of the real-time sound score.

The system included three projectors, one that projected composite, real-time images from four unique camera angles, and two that projected on the floor overlaying each other where the artist stood, creating an interactive “screen” that was mapped with the tracking camera data and the robot canvas. The floor then became a real-time visual feedback of the movements of the robot showing the path that the robot was following, shown in Figure 13. This setup allowed moments for the artist to break “eye contact” with the robot, shifting her attention and allowing more general freedom of movement in the performance without breaking the dialogue with the robot. Together, the multiple projections created an interactive environment that created an immersive space for both the performer, the operators, and the audiences, inviting the possibility for multiple perspectives on the scene as shown in Figure 14.

#### 5.2.4 Sound and Movement

The sound for the performance was generated using a contact microphone attached to the robot. The natural sound of the robot motors are fed through a filter that produces a grating, crackly sound that modulates with the movement of the robot. When the robot draws, the noise from the sound of pen making contact with the paper is amplified, calling attention to the acoustic properties of drawing. The sound is sent through two powerful speakers positioned overhead, resulting in a loud and uncanny soundscape that amplifies the presence of the robot. In addition, we used a Korg Synthesizer Monotron to add two special effects, delay and distortion, to enhance the sound at key moments during the performance. A keyboard was used to add a simple melody in at the climax of the performance. The sound was performed live by Gomez, the team engineer who is also a musician. From the outset, we knew that we wanted to explore the sounds of the robot, rather than a separate score. We conducted some early tests in the lab with contact microphones on the robot, that produced sounds that were passed through filters to generate interesting effects. This approach was inspired by previous work with contact microphones on robots and also work by Schacher and Wei (2019) that mapped brush gestures in Chinese calligraphy with sounds processes during a live performance with two performers on a shared canvas (Schacher and Wei, 2019). Our explorations revealed that we can use the amplified signal from contact microphones to achieve sonification of movement without any synthesis techniques. The performer’s body movements would facilitate the creation of mechanic sounds, strengthening the robot’s presence and also making clear to the audience the connection between the performer’s movements and the robot’s motions that produced the drawing.

### 5.3 Video Cue Recall

We conducted a video cue recall session following the performance. The topics of the conversation were not limited to the performance. Rather, the video was used as a baseline for generating a discussion about various aspects of the collaboration, including the initial research stages. Thus, the video session evolved into a semi-structured interview. We attempted to steer the conversation back to the performance, which played continually on a loop throughout the interview. We sometimes paused the playback, either slowing down or speeding up sections to focus on specific moments and review them. Loosely following the principles of thematic analysis, we identified three themes from the session: Interaction as Game, Improvisation as Dialogue, and Embodiment. Considerable discussion was given to possibilities for revising the performance or expanding it in other domains. We summarize these three themes with the view that they might be useful for facilitating creative expression for other artist/researchers interested in human-robot collaboration.

#### 5.3.1 Interaction as Game

Reviewing the performance, Rizzo described her interaction with the robot both during the performance and during the workshops. Gomez, Pekarik and Jochum were impressed by Rizzo’s candor when expressing her lack of enthusiasm for the original drawing software introduced during Workshop #1 (Pedersen et al., 2020). She expressed her dissatisfaction not only with the artistic quality of the drawings, but primarily because she didn’t see any possibility for real interaction with the system: “The very first drawing program involving robots that you presented to me was totally uninteresting. I was really in doubt about how it could be useful to me as an artist. This machine that is making (and I’m sorry to say it) but very stupid, simple and ugly drawings. And I was like, well, “How useful is it for me?” and “How entertaining is it for people looking at it?” “How interesting is it for artists?”” Rizzo elaborated that while the interaction with the first software program describe in Section 4.2 was centered on a simple guessing game (image recognition), the EMCAR tool allowed her to develop a more complex game that was open-ended and playful, thereby giving her more possibilities to explore as an artist: “If a person is looking at a robot drawing an elephant, people might say, ‚Ah, it kind of looks like an elephant.’ Or ‚Yeah, it kind of looks like an animal.’ And the game finishes there. To attract interest from people, you have to start a game. And the game finishes the moment people realize what the robot is drawing. There is nothing else to guess, there’s nothing else to see, or to imagine. You don’t want to discover more. So I think that we should leave the game a bit more open and unclear, open to investigation and imagination, so the interest of the people stays high, at least for a longer time. And with this one, I think it’s easier to keep it open for longer time. And also, it’s more interesting because every single person will see different things and will be inspired in different ways.” Rizzo also referred to the entire performance situation as a type of game, this time invoking the suspension of disbelief that is intrinsic to all theatrical productions: “There was this game (that obviously we know was fake), but there was this game that it was looking like the machine was still expressing itself, even though it was following me. And in that way, I feel inspired and I feel that I could work with it.”

#### 5.3.2 Improvisation as Dialogue

When asked about generating improvisation during the performance, Rizzo referred to improvisation as a kind of dialogue centered on nonverbal communication: “For improvisation as a solo performer, I believe you follow an inner path. So there is a dialogue inside of you. And for the dialogue you just decide who you are talking with. And sometimes you’re talking with your audience, sometimes you are just talking with yourself, in this case, sometimes I was “talking” with the robot. But you decide where to go. You decide which language you want to talk. You decide if you want to stay silent. You decide if you want to laugh or if you want to scream. So as a solo performer, life is easier. If you are in a group, you constantly have to deal with the others, to consider the others, look at the others, listen to the others. That is why improvisation in a group can be so difficult. Sometimes you lose inner concentration, you lose the inner peace that allows you to be clear in your intentions, in your dialogue intentions, what you want to say. Because a performance is nothing but a speech. You are saying something. And if you are in a group it is very difficult. That is why normally group improvisation, they have kind of boxes, or boundaries, or pre-decided limits like, if all of the sudden one person is doing this, then everybody follows that.”

#### 5.3.3 Embodiment

When asked about working with EMCAR, Rizzo described the software presented in the early workshops, which used pre-loaded images and didn’t allow for the ability control the robot in real-time. “I remember when I arrived in the lab, and you presented me with the robot that drew from files that you physically put in the computer, and I saw how the robot just recreated the drawing in a very precise and ugly way. I thought, “You know that we have printing machines, right?” Obviously I’m not trying to take down all the work that was behind it, but as a person that doesn’t know anything about programming, I believe that others would also think the same thing that I did.” Without being prompted, Rizzo compared the first software program to her experience working with EMCAR, beginning with the tablet: “So now you have a machine that I think any other person might find interesting. Because, again, we have inputs that we can put through the tablet [..] so it’s not drawing by itself, but it’s drawing through the hand of another person, and creating amazing landscapes for performers.” Rizzo was also excited about the expanded possibilities offered by the puppeteering function: “Even a robot that is a performer itself, because of the puppetry movement, that is actually an awesome thing that I really got inspired by. I thought, “Oh my God, you could do so many things with that!” And again, you also showed that it was very interesting for people to try it out. Through the puppetry movements, people also realize better how to express themselves. Like, if I want to express anger, what should I do in order to be simple? If I want to express sadness, what should I do? So people also realize more how to express those feelings through clear movements made by a machine that is absolutely without personality—no feeling, no facial expression—just a machine. The robot doesn’t even have a fake face, it doesn’t even have eyes. But still, with the right movements, that machine is alive and you turn it into a sad machine, an angry machine, a happy machine. For me, on the inside, it was very important because I was guided by the robot, but I also felt that I was guiding the robot.”

## 6 Discussion

Our project began with a custom software tool that allowed for collaborative drawing with a robot. The first drawing tool was rather naive, and the initial work did not include any collaboration with artists. We assembled and engaged a transdisciplinary team of researchers, including a professional artist trained in classical painting, dance, and circus performance, to explore the creative potential of human-robot interaction. We did not have a predefined goal or research question at the outset. Instead, we proceeded from an arts-led, practice-based research perspective to explore possibilities for human-robot creativity. Our motivation was to identify creative processes associated with visual art and understand where and how a robot might meaningfully intervene in these processes to support human creativity. When we began our project, we had no idea that we would end up making a dance performance nor did we have an idea of what tools would be necessary to make that performance realizable. This is evidence for how arts-led, practice based research can facilitate discovery-oriented behavior through discovered-problem situations. (Csikszentmihalyi and Getzels, 2014). The tools we developed were those that the artist needed, born out of exploratory practice and the textility of making and not from some preconceived idea that originated in the engineer’s or the artist’s head. The focus on process-led discovery also called attention to the dynamic relations between artistic team, tools, and materials, and eventually the audience. In the systems theory model, creativity is not an attribute inherent to a product or artefact, but depends on the effect it is able to produce in others: “What we call creativity, then, is a phenomenon that is constructed through an interaction between producer and audience” (Csikszentmihalyi, 1998, p.314). On the most basic level, our project demonstrated how the robot as an interactive system came to be regarded as creative because the performer shifted in her response and reaction to it through a constructivist approach. Through the tools and performance, Rizzo gradually came to regard the system as interactive, as something that she could actually work with. Rizzo’s characterisation of both the performance and the interaction with the robot as improvisatory and dialogic echoes Ingold’s concept of creativity as a becoming process that brings together diverse materials by “combining or redirecting their flow in the anticipation of what might emerge” (Ingold, 2009, p.94). The dialogue that emerged was not only between the performer and the robot, but involved the performance environment (projected light that animated the floor in response to the performer’s movements), materials (brushes and ink, canvas), sound, and the audience. Like Simondon’s technical ensemble, Rizzo became a kind of conductor during the performance, coordinating the action and network of tools and materials as well as the activities of the other members of the artistic team seated onstage at their computers.

Observing her performance with robot, Rizzo described her interaction with the robot as a kind of game. The strategy of game as a concept for designing has been studied in the context of interactive media art, (Kluszczynski, 2010), but has yet to be taken up in human-robot interaction. According to Kluszczynski, the Strategy of Game organizes events and outcomes that emerge from the interaction itself. A basic characteristic of this strategy involves a specific task to be performed, where each participant has access to the rules and tools of the game and a certain amount of space. The strategy of game differs from games because it draws the attention of participants “not only toward the tasks that are outlined, but also toward the interaction’s course, its architecture, relations between the game’s structure and its properties, and also the other discourses included in the event.” Art works that utilize the strategy of game “place in the discursive opposition not only the player and the game, but also the process of playing, in this way gaining the possibility to make all these aspects of the game and the game world as understood generally debatable” (Kluszczynski, 2010, p.8). Another feature of this strategy is that it allows for the possibility to approach metadiscursive issues that are not directly connected with the game or outcome, thereby enabling the artwork/interaction to develop discourses within its own structure that are critical toward the game/task. One can imagine approaching interaction design and interfaces for human-robot interaction that allow for this kind of critical engagement. The result could be an interface that aims at intuitive, natural interaction while making clear the underlying logic and limitations at work in the system.

Artists’ experimentation with conceptual and material representation plays an integral part in the creative process (Dahlstedt, 2012). The artist can explore more intuitively the possibilities of what a robot can do when the system offers interaction in a natural way that echoes her process of making, not only the outcome or product. Embodied interaction is an interaction with technology that offers an opportunity to interact with the system naturally. As Dahlstedt (2012) points out, new ideas are more likely to emerge from the iterative process where the artist is directly engaged in a dialogue between conceptual and current material manifestation. The important aspect is that the material offers the possibility for this type of this communication—like a sculptor working with marble. The advantage of a system that uses embodied interaction is that the artist is empowered to refine possible conceptual and material spaces with more ease. Working through the material’s resistance can challenge the artist’s desire to shape the form. As mentioned in Nake (2012), artistic expression requires that the artist finds creative ways to work with or through resistance of the material, in order to shape it. Physically, materials occupy a spectrum of resistance. According to Dahlstedt (2012), tools offers navigation in the limitless space of intrinsic material possibilities, but only along the paths that the tools provide. If navigating those paths can become intuitive, the process of exploration is accelerated, which results in an artist’s expression. In this sense, our program stands apart from algorithmic and digital art. Even though we were working with software, the nature of the system, through its embodied interaction capabilities, influenced how we navigate that space of possibilities. For instance, programming robots using embodied control allows for greater accessibility that makes working with robots more accessible for people without engineering or computer science backgrounds. We took inspiration from puppetry, where traditional puppeteers enjoy immediate feedback by working directly with the material/puppet. This creative process typically depends on immediate response and force feedback of the animated object, which help the artist to design choreography intuitively and create expressive movements/animation. For artists not used to working with technology, working without this direct feedback can be challenging. The ability to control the robot with her body or through a stylus gave Rizzo a completely new perspective on the machine: “It’s a completely different machine. Now, it’s a colleague, it’s a pal that I would like to work with. I’m looking forward to work again with it.” The importance of embodied computing and its relevance for meaning making and perception is well documented (Wainer et al., 2006; Sheets-Johnstone, 2011; Fdili Alaoui et al., 2015).

Recent scholarship in HCI, informed by Disability Studies and critical feminist scholarship, has highlighted the ways in which the conventional approaches to design for embodied interaction are highly problematic (Giaccardi and Karana, 2015; Shildrick, 2013). As Katta Spiel notes in (Spiel, 2021), “bodies and how we design for them are products of social norms,” and these norms contain dangerous adverse consequences for bodies and people that do not fit readily inside these normative categories. Much of HRI and literature on embodied interaction equate being human with white, male, non-disabled bodies. The implicit Western male whiteness contained in the conceptualisations and artefacts in the field of embodied computing are more than mere blindspots, they materialize and encode bias and do not account of the experiential differences in lived embodiments of women, BIPOC or people with disabilities. The result is that practices in the field of embodied computing fail to account for the “axes of oppression” that reify certain forms of power, rendering it all but impossible to rethink or design for bodies outside of normative categories. Unfortunately, critical inquiries like Spiel’s do not feature prominently enough in HCI or HRI research, although there are promising signs that this practice is beginning to change. Design for embodied interaction that allows for plurality and difference of human embodiments can and should be considered when designing embodied controllers or devices. In our project, we focused primarily on developing tools for Rizzo that would not require programming skills or understanding the underlying logics of the system. Rizzo is a non-disabled dancer with decades of training in somatic and dance practices. We were attentive to the lived, bodily experience of the artist working with the tools and the difference in how she encountered tools in her atelier versus the tools in the lab. Our intention was not to encumber Rizzo with gadgets or tools, but to provide an embodied experience that was reminiscent of the tools and the way she worked with those tools in her own visual art practice. The initial experiments with the drawing tablet and stylus were familiar to Rizzo from her work with computer drawing tools as a children’s book illustrator. However, the drawing technique for controlling the robot motion did not do much to inspire her. It wasn’t until Rizzo was presented with the motion tracking technology and moving projections on the floor, which allowed her to directly observe the link between her physical movements and the movement of the robot, that she began to feel inspired to work creatively and empathically with the robot.

Gemeinboek (after Dautenhahn) problematizes the notion of corporeal empathy and embodied interaction for designers: how does one design for embodied interaction when there is no such thing as “natural interaction”? (Gemeinboeck, 2021) As shown by Fdili Alaoui et al. (2015) and Gannon (2018), human-centered interfaces can enhance, augment, and expand human capabilities through bodily extensions or worn prosthesis. Typically these devices rely on sensors or other wearable controllers that control or direct the movement of the robot, usually through remote tele-operation. Such devices can be read as prosthesis. Disability studies scholar Margrit Shildrick has advanced critical perspectives that link technologies and devices with affective experiences and subjectivity. Shildrick’s notion of embodiment and embodied interaction explores the “affective significances of prosthesis and devices that transform the body, demonstrating how corporeal transformations can work to undo the coventional limits of the embodied self” (Shildrick, 2013). She identifies in prosthetic devices a potential for a “celebratory reimagining of the multiple possibilities of corporeal extensiveness” (Shildrick, 2013, p.271). While the tracking technology we experimented cannot be called a prosthesis, the fact that Rizzo was able to control the robot and produce two sets of drawings—one on the canvas of the floor through projected light, and the other through the robot and the canvas on the desk, we can read the entire system as a kind of technical ensemble, or a type of prosthesis that expanded the conventional limits of Rizzo’s body and triggered her imagination. The convergence of artist-tool-material-space brought about a new corporeal configuration that begin to make possible a creative re-imagining of alternate forms of embodiment and artistic expression (Donnarumma, 2020). It is also interesting to note how the experience of working collaboratively and creatively with the artist impacted the perspectives of the other members of the research team in ways we could not have imagined beforehand. For example, reflecting on the workshops, Gomez (an engineer) commented that the entire experience changed his perspective on how he would approach research problems in the future. For example, his next project involves using a CNC machine to carve mortar for facades. He remarked that before beginning development on that project, he would begin by exploring the technique by hand, in order to gain an embodied understanding of working with and through the materials. Our arts-led, practice-based investigation reconfirmed the necessity of tactile and sensuous exploration and knowledge of materials, knowledge that has long been considered tangential to cognitive theories of creativity, but deeply entangled with creative artistic practice. We learned that embodied exploration of material was not only important for the artist (Rizzo) when working with robots, but also for programmer (Pekarik) and engineer (Gomez) responsible for designing the interactive systems. During the weekly drawing and painting workshops, the research team experimented together using different tools and collaborating on a shared canvas. The sustained interaction allowed the partners to delve more deeply into each other’s world and material practice, providing us with an embodied understanding of artistic processes and tools that we would not otherwise have access to. Positioning the canvas on the ground and collaborating together on a shared canvas both defamiliarized the activity of drawing and invited another way of knowing and relating to materials and to one another.

Thinking through the material is key when designing tools or systems. Tools are, of course, extensions of the artist, although the artist does not necessarily need to be able to produce the tool in order to utilise it. Engineers, on the other hand, are specialized in creating tools that allow others to explore the material creatively. The sustained interaction among the members of the research team generated a bond, that through iteration grew stronger and resulted in embodied knowledge exchange and appreciation of different perspectives. The different workshops helped to generate this bond and to find common ground where the desires and expectations of the artist and engineers met from functional, reliable and safe perspectives. Reflecting on the co-creative aspect, Pekarik expressed that understanding the intrinsic motivation of the drawing activity as a communication process between artist and the material helped him to prioritize design decisions towards embodiment qualities. The authors all agree that this close collaboration enlightened the best practical possibilities and positively influenced the research outcomes. Although the process resulted in new software and hardware tools for artists to work with, to regard these tools as creative in themselves would be shortsighted. These creative outcomes are not finished products, but artefacts that open up new possibilities for creative exploration across new topologies. Rather than products that signal creative outcomes, they function as material evidence for creative processes. We plan to continue working with these tools and processes to develop more diverse tools for the artist-robot team to explore, both in the laboratory and in the atelier. Current research exploring expressive robot animations by Pakrasi et al. (2018) and real-time interaction with “live” algorithms in performance by Blackwell et al. (2012) indicate possible future directions. Through our investigation, we widened our own conceptual models of human-machine interaction and co-creation. Art involves generative processes that require negotiation and interaction with physical materials and tools for art making. Artistic and creative processes are not confined to human-tool interaction, producer-audience relations, or product-audience judgements. Artistic creativity is capacious: it extends to the environment and involves an entire network of physical and digital objects, organic and inorganic, artificial and natural, entangled in a field of relations that is continually shifting, recompiling, and interweaving between physical and virtual spaces, through planned and unplanned actions. If live performance is where the planned and the unexpected meet, we can imagine no better site for creatively exploring new possibility spaces for robotics and human-robot interaction.

## 7 Conclusion

Typically, problems in robotics take the form of presented problem situations, where the problem and tools for solving the problem are known at the outset. Our exploratory, trandiscplinary research began with a different intention: utilizing creative methods, we generated discovered-problem situations to generate new ideas and approaches for designing interactive systems and human-machine interaction. Rather than focusing on a robot that could produce artistic outcomes, we focused on drawing as an activity that could help us explore more deeply “the itinerant, improvisatory and rhythmic qualities of making” (Ingold, 2009, p.99). Drawing is intrinsically dynamic and temporal, and can be understood as a process of becoming, rather than being: “You cannot be a mountain, or a buzzard soaring in the sky, or a tree in the forest. But you can *become* one, by aligning your own movements and gestures with those of the thing you wish to draw. […] As with the mountain path, the buzzard’s flight or the tree root, the drawn line does not connect predetermined points in sequence but ‚launches forth’ from its tip, leaving a trail behind. […] It has no end point: one can never tell when a drawing is finished” (Ingold, 2009, p.99). Our project demonstrates the possibilities of reimagining human-machine collaboration and technical ensembles. We found strong links between artistic creativity and discovered problem-solving processes. Iterating and developing ideas in an open-ended (as opposed to predefined) manner altered, evolved, and expanded the outcome of our creative process in ways that we could not have anticipated. This process was reminiscent of what Ingold calls “looping” - the processes of an artist working directly with tools and materials in a dialogic manner. The concept of dialogue emerged as a salient feature for both improvisation in performance, and the human-robot interaction during the collaborative drawing sessions. Transdisciplinary research facilitates creative processes between humans and machines, allowing the interactions to take shape with and through materials in dynamic and collaborative encounters.

## Data Availability

Publicly available datasets were analyzed in this study. This data can be found here: https://github.com/marospekarik/ur-interface.

## Footnotes

1 Creative Confession, Klee (2013).

2 The complete code for the EMCAR system can be found in the following repository: https://github.com/marospekarik/ur-interface.

3 Video of the In-Pulse performance at https://youtu.be/0nMKvoos6TQ

4 Video of the full performance *If/Then* available at https://vimeo.com/491681339
